# Accumulation of Wheat Phenolic Acids under Different Nitrogen Rates and Growing Environments

**DOI:** 10.3390/plants11172237

**Published:** 2022-08-29

**Authors:** Wenfei Tian, Fengju Wang, Kaijie Xu, Zhaoxing Zhang, Junliang Yan, Jun Yan, Yubing Tian, Jindong Liu, Yan Zhang, Yong Zhang, Zhonghu He

**Affiliations:** 1 National Wheat Improvement Centre, Institute of Crop Sciences, Chinese Academy of Agricultural Sciences, Beijing 100081, China; 2 International Maize and Wheat Improvement Center (CIMMYT) China Office, Chinese Academy of Agricultural Sciences, Beijing 100081, China; 3 Institute of Cotton Research, Chinese Academy of Agricultural Sciences, Anyang 455000, China

**Keywords:** functional wheat, *trans*-ferulic acid, nitrogen management, environment interaction, antioxidant

## Abstract

The health benefits of whole wheat grains are partially attributed to their phenolic acid composition, especially that of *trans*-ferulic acid (TFA), which is a powerful natural antioxidant. Breeders and producers are becoming interested in wheat with enhanced health-promoting effects. This study investigated the effects of different nitrogen (N) application rates (0, 42, 84, 126, and 168 N kg ha^−1^) on the phenolic acid composition of three wheat varieties in four locations for two years. The results indicate that the different N rates did not affect the TFA concentration but that they significantly affected the concentrations of *para*-coumaric acid, sinapic acid, and *cis*-ferulic acid in the wheat grains. A statistical analysis suggested that the wheat phenolic acid composition was predominantly determined by wheat variety, though there existed some interaction effect between the wheat variety and environments. The TFA concentration of the variety Jimai 22 was generally higher (with a mean value of 726.04 µg/g) but was easily affected by the environment, while the TFA concentration of the variety Zhongmai 578 (with a mean value of 618.01 µg/g) was more stable across the different environments. The results also suggest that it is possible to develop new wheat varieties with high yield potential, good end-use properties, and enhanced nutraceutical values.

## 1. Introduction

Wheat is a staple food worldwide [1]. Variety, growing environment, and the application of nitrogen fertilizers are important factors affecting wheat yield, protein content, and end-use quality [2,3,4]. Wheat is not only an important source of energy but also a good source of functional ingredients [5,6,7]. The consumption of whole wheat products has been associated with reduced risks of chronic diseases, including type II diabetes, obesity, and some types of cancer [8,9]. These health benefits can be partially attributed to the phytochemicals in wheat grains [10,11]. *Trans*-ferulic acid (TFA), a powerful natural antioxidant, is the most predominant phytochemical in whole wheat [12]. Most wheat phenolic acids are ester-linked to cell wall (poly)saccharides [13,14,15]. The health-promoting potential and possible mechanism of wheat phytochemicals have been intensively characterized and summarized in some literature reviews [16,17,18,19]. Driven by consumers’ desires for healthier food ingredients and final products, a higher content of phytochemicals, especially phenolic antioxidants, is becoming another important parameter in evaluating wheat quality [20,21,22].

In recent years, studies have investigated the factors determining phenolic acid composition in whole wheat grains. Similar to other agronomic traits, wheat variety is an important factor affecting phenolic acid composition [23,24,25,26,27]. For example, the HEALTHGRAIN Diversity Screen reported a range of >3.5-fold across the concentration range for total phenolic acids in 135 winter wheat varieties [24]. Several studies have reported that some wheat varieties accumulated higher concentrations of phenolic acids in dry and hot cultivation years [28,29,30]. It is well known that nitrogen fertilizer is a very important factor influencing the yield and quality of wheat grains. Previous studies have also evaluated the effects of nitrogen fertilization and its interaction with other factors on wheat phenolic composition. In a previous study, it was reported that increased N application (from 180 to 300 kg N ha^−1^) led to increased concentrations of insoluble ferulic acid and vanillic acid [31]. However, this range of nitrogen application rate is much higher than that of farmers’ general practices. Furthermore, only one wheat variety was investigated, and the effect of nitrogen fertilizer may be dependent on wheat variety. Our previous study found an interaction effect of N and sulfur (S) on the concentration of *trans*-ferulic acid: at S = 0, an increased N rate (from 56 to 146 kg N ha^−1^) led to an increased TFA concentration, and at S = 22 kg ha^−1^, a different N rate did not influence the TFA concentration [28]. However, a following study suggested that intensified field management practices, including the increased application of nitrogen fertilizer, fungicide, and micronutrients, led to a decreased concentration of TFA [32]. However, it was unknown which factor of the intensified management practices was the major contributor to the decreased TFA. In summary, previous studies have usually contained several factors that may influence TFA concentration, and the effect of the N rate is still not clearly understood. A study mainly focusing on the N rate and wheat phenolics in different varieties is still needed.

Our hypotheses were that different N rates are not a major factor determining TFA concentration and that wheat phenolic acid composition is mainly affected by wheat variety. Therefore, the objectives of this study were to (1) investigate the effect of different N rates (N0, N42, N84, N126, and N168 kg ha^−1^) on wheat phenolic acid composition and to (2) assess the most important factor affecting wheat phenolic acid composition and its interaction with other factors. Three elite Chinese wheat varieties were harvested in eight natural environments (over two years, at four locations each year). To the best of our knowledge, this is the most thorough study investigating the effect of N fertilization on wheat phenolics in terms of the number of N levels, wheat varieties, and different environments. For all three varieties, under the conditions of eight environments (a total of 24 conditions), the effect of N fertilization was consistent and barely interacted with other factors. The reliable and clear results of this study may help to elucidate the effect of N rate on the accumulation of wheat phenolic antioxidants. This information may also be instructive to wheat production, aiming at producing grains with enhanced natural antioxidants.

## 2. Materials and Methods

### 2.1. Plant Materials and Field Experiments

Three elite Chinese bread wheat varieties, namely, Zhongmai 578, Jimai 22, and Zhongmai 255, were investigated in this study. Zhongmai 578, developed from Zhongmai 255/Jimai 22, was released in the northern and southern parts of the Yellow and Huai Valleys covering the provinces of Henan, Shaanxi, northern Anhui and Jiangsu, Shandong, Hebei, and central Shanxi, as well as southern Xinjiang, in 2020, 2021, and 2022. It is becoming a leading variety, with a sowing acreage of around 340,000 ha in the 2021–2022 harvesting season, due to its high yield potential, superior pan bread-making quality, good disease resistance, tolerance to high temperatures during the grain-filling period, and early maturity, as well as broad adaptation. Zhongmai 578 is also widely used as a parent in various breeding programs across China. Jimai 22 has been the milestone variety in the northern part of the Yellow and Huai Valleys for the last 15 years, and it has a high and stable yield, and broad adaptation. Zhongmai 255 was released due to its superior qualities for both pan bread and Chinese noodles.

Field experiments were performed during the 2019–2020 and 2020–2021 wheat-growing seasons at four locations, namely, Anyang, Henan province (36°05’45.60” N 114°22’58.01” E); Huaibei, Anhui province (33°58’27.98” N 116°47’30.01” E); Liaocheng, Shandong province (36°27’21.46” N 115°58’39.58” E); and Weifang, Shandong province (36°42’36.00” N 119°06’6.98” E). Urea (NH2)_2_CO (N% = 46.67%) was used as the nitrogen (N) fertilizer. For each wheat variety, the field experiments included five urea application rates: 0 (N content = 0), 90 (N content = 42), 180 (N content = 84), 270 (N content = 126), and 360 (N content = 168) kg ha^−1^, designated as N0, N42, N84, N126, and N168, respectively. Each treatment comprised three replicates. Other field management practices were the same for all locations. After harvest, wheat seeds were stored at 4 °C until further analysis.

### 2.2. Reagents and Chemicals

Analytical standard phenolic acids (4-hydroxybenzoic acid, vanillic acid, *para*-coumaric acid, *trans*-ferulic acid, and sinapic acid) were purchased from MACKLIN (Shanghai, China). Other general chemicals and ultra-performance liquid chromatography (UPLC) mobile phases (water with 0.1% trifluoroacetic acid and acetonitrile with 0.1% trifluoroacetic acid) were purchased from Thermo Fisher Scientific (Waltham, MA, USA).

### 2.3. Extraction of Total Esterified Phenolic Acids

The extraction of esterified phenolic acids was performed according to our previous studies [28,32]. In brief, 1 g of whole flour was hydrolyzed with 10 mL of 2M NaOH under nitrogen for 3 h. The mixture was then acidified to pH = 2 with concentrated HCl and extracted three times with ethyl acetate. The combined organic phase was evaporated to dryness, re-dissolved using 2 mL HPLC-grade methanol, and filtered through a 0.45 µm filter. All extracted samples were kept at −20 °C and analyzed within 2 days.

### 2.4. UPLC Analysis of Phenolic Acid Composition

The phenolic acid composition was analyzed using an UPLC-PDA system from Waters Corporation (Milford, MA, USA) with an ACQUITY UPLC BEH C18 (2.1 mm × 50 mm) column. The UPLC gradient protocol was programed according to our previous report with some minor modifications [33]. In brief, the mobile phase A was HPLC-grade water containing 0.1% trifluoroacetic acid, and the mobile phase B was HPLC-grade acetonitrile containing 0.1% trifluoroacetic acid. The mobile phase flow rate was 0.4 mL/min, and the percentage of mobile phase B was kept at 6% from 0 to 1.0 min, which then increased linearly to 14% from 1.0 min to 3.0 min and increased linearly to 16% from 3.0 min to 5.5 min. The column was re-equilibrated with the original mobile phase condition (A = 94%, B = 6%) for 3 min between each injection. Each phenolic acid was identified by a comparison with the retention time of the corresponding analytical standard and quantified using an external calibration curve at 280 nm. Due to the lack of analytical standard, *cis*-ferulic acid was tentatively quantified using the external calibration of *trans*-ferulic acid.

### 2.5. Statical Analysis

The raw experimental data (concentration of each phenolic acid) are expressed as mean ± standard deviations from three biological replicates. Further data analysis was performed using the PROC GLIMMIX method in SAS version 9.4 (Cary, NC, USA). Year, location (loc), wheat variety (V), N rate, and their interactions were considered as fixed effects. Replicate (rep), rep(year), rep(loc), N*rep(year), and N*rep(loc) were considered as random effects.

## 3. Results and Discussions

In this study, six monomeric phenolic acids, namely, 4-hydroxybenzoic acid, vanillic acid, *para*-coumaric acid, *trans*-ferulic acid, sinapic acid, and *cis*-ferulic acid, were detected in the extracts of whole wheat flours. The concentration of TFA ranged from 491.40 ± 28.20 to 831.25 ± 3.80 µg/g whole wheat flour (WWF). The concentrations of 4-hydroxybenzoic acid, vanillic acid, and *para*-coumaric acid were found to be at lower levels, usually less than 30 µg/g WWF. The concentration of *cis*-ferulic acid and sinapic acid ranged from 38.42 ± 0.18 to 103.38 ± 2.19 µg/g and from 11.88 ± 0.65 to 98.97 ± 6.41 µg/g WWF, respectively. These results are comparable to those of previous studies [17,19,25].

### 3.1. Summary of The Analysis of Variance (ANOVA)

The result of ANOVA is tabulated in Table 1. The effect of the wheat variety (V) was found to be significant (*p* < 0.001) for all the phenolic acids. The effects of location (L) and harvest year (Y) were also significant for most types of phenolic acids detected in this study. The effect of L*V*Y was also significant (*p* < 0.001) for TFA and for most other phenolic acids. These results agree with the previous consensus that both wheat variety and environment affect the phenolic acid composition in wheat grains [29,31,33,34]. The effect of the nitrogen (N) application rate was not significant (*p* > 0.05) for the concentration of TFA or the predominant phenolic compound in whole wheat, but it was significant (*p* < 0.001) for *para*-coumaric acid, sinapic acid, and *cis*-ferulic acid. L*N was significant (*p* < 0.05) for TFA. Since the major objective of this study was to investigate the effect of the nitrogen application rate on TFA, these results are discussed in detail in the following section.

### 3.2. Effects of L, V, Y, and Their Interactions on TFA Concentration

**Figure 1:**
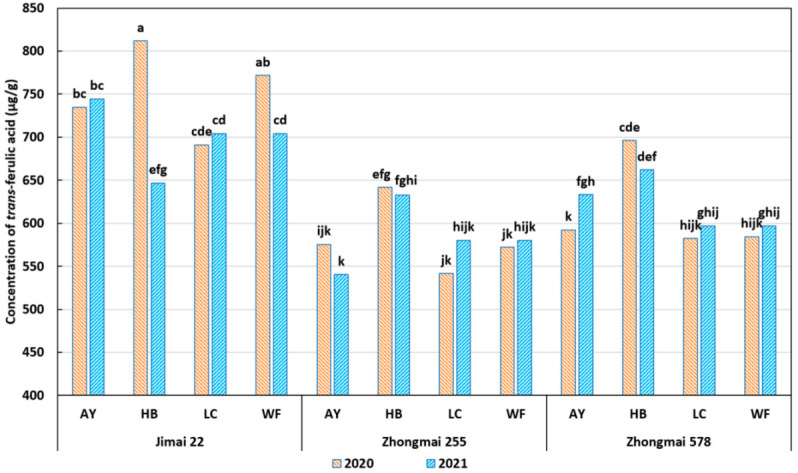
Effect of L*V*Y on concentration of *trans*-ferulic acid. L: location; V: variety; Y: year; AY: Anyang; HB: Huaibei; LC: Liaocheng; WF: Weifang. Values with no letter in common are considered significantly different (*p* < 0.05).

As shown in Table 1, the effects of L and V on TFA were significant (*p* < 0.001). Overall, across all environments, the variety Jimai 22 had the highest mean concentration of TFA (726.04 µg/g), followed by Zhongmai 578 (618.01 µg/g) and Zhongmai 255 (583.07 µg/g). Regarding the location effect, in both 2020 and 2021 years, the wheat produced from Huaibei had the highest mean concentration of TFA (681.83 µg/g), and the wheat from Liaocheng had the lowest concentration of TFA (616.03 µg/g). The interaction effects between L, V, and Y are plotted in Figure 1. Though the variety Jimai 22 generally contained a high concentration of TFA, this concentration was easily affected by the different environments. In the Huaibei location, the TFA concentration of Jimai 22 decreased significantly (*p* < 0.05) from 811.73 µg/g in 2020 to 646.48 µg/g in 2021. Similarly, in the Weifang location, the TFA concentration of Jimai 22 decreased significantly (*p* < 0.05) from 771.56 µg/g in 2020 to 704.24 µg/g in 2021. In contrast, the TFA concentration of Zhongmai 578 was more stable across the different environments. This result suggests that Jimai 22 could only accumulate more ferulic acid under certain environments and that the three varieties might differ in the pathway of TFA accumulation.

Several previous studies have reported that dry weather condition (drought stress) during the grain-filling stage might enhance the TFA concentration in wheat grains [28,29,30]. In this study, the four locations had similar temperatures during the wheat growth period (Table 2). In May and June of 2019–2020, Huaibei was the location with the most precipitation, and Liaocheng was the driest location with the lowest precipitation. However, the samples from Huaibei contained the highest TFA, while the samples from Liaocheng had the lowest TFA. Similarly, considering the Huaibei location, the growth period of 2019–2020 had more rainfall than that of 2020–2021, but the wheat grains from 2020 had more TFA than the grains from 2021, especially in the case of Jimai 22. These results suggest that drought stress was not the predominant factor among all the environmental factors determining TFA concentration. In summary, the final concentrations of TFA in the wheat grains were determined by the interaction effect between the wheat variety and environmental factors, including, but not limited to, the different locations and harvest years. This result is consistent with a recent study that also reported significant effects of variety and location on wheat phenolic acid composition [35].

### 3.3. Effect of Nitrogen Application on Wheat Phenolic Acid Composition

**Figure 2:**
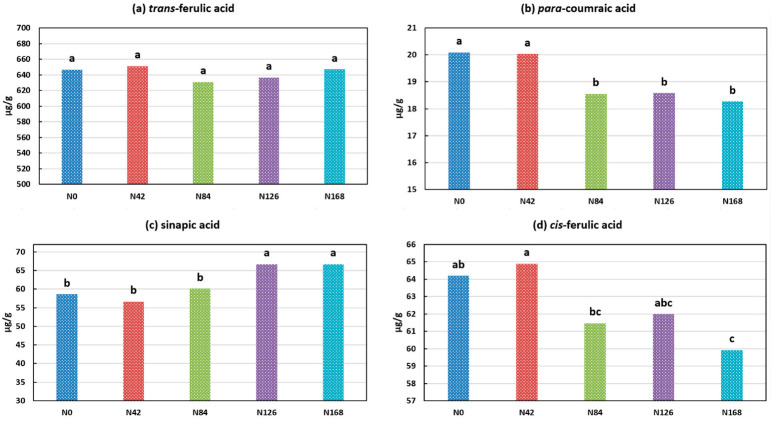
Effect of different nitrogen rates on phenolic acid composition (**a**) *trans*-ferulic acid; (**b**) *para*-coumaric acid; (**c**) sinapic acid; (**d**) *cis*-ferulic acid. Within each figure, values with different letters are considered significantly different (*p* < 0.05).

**Figure 3:**
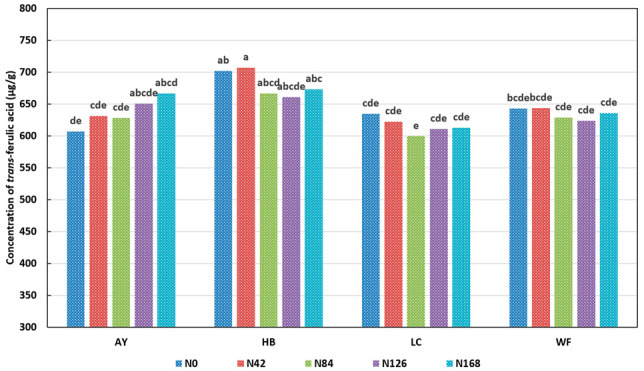
Effect of N*V on concentration of *trans*-ferulic acid. N: nitrogen rate; V: variety; AY: Anyang; HB: Huaibei; LC: Liaocheng; WF: Weifang. Values with no letter in common are considered significantly different (*p* < 0.05).

One major goal of this study was to elucidate the effect of different nitrogen fertilizer (as urea) rates. The single effect of N rate on the concentration of individual phenolic acids is plotted in Figure 2a–d for *trans*-ferulic acid, *para*-coumaric acid, sinapic acid, and *cis*-ferulic acid, respectively. In Figure 2a, it seems that the wheat grains contained relatively more TFA at either a very low (N0 and N 42) or a very high N (N168) level. However, this difference was not significant at a confidence level of *p* < 0.05. However, the effect of N rate on other major phenolic acids was significant (*p* < 0.05). The L*N interaction for the TFA concentration was significant (*p* < 0.05), and it is plotted in Figure 3. There were some differences under different N rates and at different locations. In Anyang, the TFA concentration increased with an increase in the N rate. In the other three locations, the TFA concentration generally decreased first and then increased along with the increased N application. However, most of these changes were not significant at a confidence level of *p* < 0.05. Previously, we reported that, under sulfur-sufficient conditions, increased N application had no effect on wheat *trans*-ferulic acid concentrations [28]. The result from this study confirms our previous finding. Taken together, these two studies comprise four growth years, various locations, and representative wheat varieties in both China and the United States. Based on these results, it is concluded that the nitrogen application rate did not affect the concentration of *trans*-ferulic acid in the wheat grains. A similar result has also been previously reported [34]. This observation can be partially explained by a previous study, which reported that different N rates did not affect the transcriptions of phenylalanine ammonia lyase (PAL) or cinnamate 4-hydroxylse (C4H), the key enzymes of the phenylpropanoid pathway [36].

In Figure 2b,d, an increased N level led to decreased concentrations of *para*-coumaric acid (from 20.09 µg/g in N0 to 18.27 µg/g in N168) and *cis*-ferulic acid (from 64.19 µg/g in N0 to 59.91µg/g in N168). In contrast, an increased N level generally led to an increased concentration of sinapic acid, from 58.59 µg/g in N0 to 66.68 µg/g in N24. Though these changes were considered statistically significant, the changes in the absolute values were not huge. An increased N rate led to opposite changing trends for the different phenolic acids. This result suggests that there existed a hidden metabolic pathway for the different phenolic acids that was affected by the different N rates.

To summarize, the data in this study indicated that the different N rates were not a major factor determining the phenolic acid composition of the wheat grains, though the effect of the N rate might be significant under the conditions of certain experimental designs and statistical methods. The concentration of phenolic acid, especially *trans*-ferulic acid, was mainly determined by wheat variety and the matrix of natural environmental factors. The content of the functional ingredients in some wheat varieties, such as Zhongmai 578, was stable across the various environments and the different nitrogen application levels. The development of a new wheat variety with enhanced health benefits seems to be a practical and promising goal for wheat breeders, as previously claimed by Shewry et al. [20].

## 4. Conclusions

This study investigated the effects of different nitrogen (as urea) application rates, growth locations, and years on the phenolic acid composition of three Chinese elite wheat varieties. Our results demonstrate that nitrogen fertilizer usage was not a major factor affecting wheat phenolic acid composition. Wheat variety was the predominant factor determining wheat phenolic acid composition. The effect of environmental factors was also dependent on the wheat variety. The concentration of *trans*-ferulic acid, a powerful natural antioxidant, in Zhongmai 578 was stable across the different environments and management practices. Future studies are necessary to understand the genetic basis and regulatory network for the production of phenolic antioxidants in whole wheat grains.

## Figures and Tables

**Table 1:** Analysis of variance (ANOVA) for phenolic acid composition of wheat varieties under the conditions of different environments.

Source of Variation	DF	HBA	VA	PCA	TFA	SA	CFA
Location(L)	3	0.15	32.76 ***	24.02 *	35.51 ***	15.25 ***	59 ***
Nitrogen(N)	4	0.48	1.13	13.02 ***	1.85	11.45 ***	7.16 ***
Variety(V)	2	22.59 ***	12.18 ***	807.49 ***	405.87 ***	59.17 ***	345.43 ***
Year(Y)	1	49.82 ***	34.03 ***	295.1 *	7.61 *	12.21 ***	177.2 **
L*N	12	1.02	0.67	3.37	2.82 *	3.25 **	1.2
L*V	6	5.59 ***	9.07 ***	5.34 ***	10.8 ***	10.47 ***	5.79 ***
L*Y	3	3.86 *	23.47 ***	46.67 ***	21.66 ***	13.68 ***	175.92 ***
N*V	8	2.01	1.23	2.38 *	2.00	2.35 *	1.07
N*Y	4	1.14	2.55	9.18 *	1.99	5.24 **	10.92 ***
V*Y	2	4.77 *	12.72 ***	27.76 ***	20.05 ***	20.09 ***	8.56 ***
L*N*V	24	0.93	0.96	1.44	1.79	2.31 **	1.19
L*N*Y	12	0.93	1.2	4.41 ***	1.38	3.18 ***	2.19 *
L*V*Y	6	2.92 *	21.25 ***	35.17 ***	9.67 ***	14.84 ***	3.93 **
N*V*Y	8	2.09 *	1.22	2.27 *	1.62	2.71 **	1.16

***, **, and * indicate significance at *p* < 0.001, 0.01, and 0.05, respectively. DF: degree of freedom; HBA: 4-hydroxybenzoic acid; VA: vanillic acid; PCA: *para*-coumaric acid; TFA: *trans*-ferulic acid; SA: sinapic acid; CFA: *cis*-ferulic acid.

**Table 2:** Temperature and rainfall conditions during the growing season of wheat. AY: Anyang; HB: Huaibei; LC: Liaocheng; WF: Weifang.

Year	Data (Month)	AY		HB		LC		WF	
		Temp (°C)	Rainfall (mm)	Temp (°C)	Rainfall (mm)	Temp (°C)	Rainfall (mm)	Temp (°C)	Rainfall (mm)
2019–2020	October	15.8	65.4	17.0	28.6	15.7	32.3	16.0	18.4
	November	9.1	0.4	11.2	9.8	9.1	31.5	9.3	14.7
	December	2.7	8.4	4.8	21.7	2.4	13.4	2.1	19.7
	January	0.8	25	2.8	75.1	0.8	24.8	1.0	27.4
	February	5.1	11.5	6.4	30.9	5.3	15.7	4.2	58.3
	March	11.8	9.1	10.7	28.1	10.9	2.7	9.7	3.8
	April	15.0	25.9	15.2	16.7	14.5	30.2	13.7	15.5
	May	23.0	70.2	22.9	30.7	22.0	27.7	20.5	99.8
	June	26.4	80.8	25.8	304.7	26.6	66.7	25.4	76.6
2020–2021	October	14.6	11.5	15.7	8.1	14.6	10.4	14.9	6.5
	November	9.1	24.2	10.4	42.1	8.9	48.9	8.8	48.0
	December	1.0	1.8	2.4	6.7	0.4	4.4	0.2	10.7
	January	1.0	0.0	2.6	6.7	−0.2	0.1	−0.5	4.3
	February	7.1	41.7	8.0	26.5	6.2	45.5	5.3	18.7
	March	10.3	16.7	10.3	55.4	10.2	16.1	9.4	33.0
	April	15.0	15.4	14.4	28.6	14.7	26.7	13.7	74.1
	May	22.0	17.4	21.2	94.9	21.4	21.8	20.2	51.3
	June	28.0	60.4	27.1	99.9	27.5	129.5	25.6	77.8

## Data Availability

The data presented in this study are available on request from the corresponding author.

## References

[1] Godfray H.C.J., Beddington J.R., Crute I.R., Haddad L., Lawrence D., Muir J.F., Pretty J., Robinson S., Thomas S.M., Toulmin C. (2010). Food Security: The Challenge of Feeding 9 Billion People. Science.

[2] Yong Z., Zhonghu H., Ye G., Aimin Z., Van Ginkel M. (2004). Effect of Environment and Genotype on Bread-Making Quality of Spring-Sown Spring Wheat Cultivars in China. Euphytica.

[3] Shewry P.R. (2009). Wheat. J. Exp. Bot..

[4] Mefleh M., Conte P., Fadda C., Giunta F., Piga A., Hassoun G., Motzo R. (2019). From Ancient to Old and Modern Durum Wheat Varieties: Interaction among Cultivar Traits, Management, and Technological Quality. J. Sci. Food Agric..

[5] He H.-J., Qiao J., Liu Y., Guo Q., Ou X., Wang X. (2021). Isolation, Structural, Functional, and Bioactive Properties of Cereal Arabinoxylan─A Critical Review. J. Agric. Food Chem..

[6] Dhua S., Kumar K., Kumar Y., Singh L., Sharanagat V.S. (2021). Composition, Characteristics and Health Promising Prospects of Black Wheat: A Review. Trends Food Sci. Technol..

[7] Ma D., Wang C., Feng J., Xu B. (2021). Wheat Grain Phenolics: A Review on Composition, Bioactivity, and Influencing Factors. J. Sci. Food Agric..

[8] Ciudad-Mulero M., Barros L., Fernandes Â., CFR Ferreira I., Callejo M.J., Matallana-González M.C., Fernández-Ruiz V., Morales P., Carrillo J.M. (2020). Potential Health Claims of Durum and Bread Wheat Flours as Functional Ingredients. Nutrients.

[9] Liu R.H. (2007). Whole Grain Phytochemicals and Health. J. Cereal Sci..

[10] Okarter N., Liu R.H. (2010). Health Benefits of Whole Grain Phytochemicals. Crit. Rev. Food Sci. Nutr..

[11] Sang S., Landberg R. (2017). The Chemistry behind Health Effects of Whole Grains. Mol. Nutr. Food Res..

[12] Tian W., Ehmke L., Miller R., Li Y. (2019). Changes in Bread Quality, Antioxidant Activity, and Phenolic Acid Composition of Wheats During Early-Stage Germination. J. Food Sci..

[13] Vaidyanathan S., Bunzel M. (2012). Development and Application of a Methodology to Determine Free Ferulic Acid and Ferulic Acid Ester-Linked to Different Types of Carbohydrates in Cereal Products. Cereal Chem..

[14] Moore J., Hao Z., Zhou K., Luther M., Costa J., Yu L. (2005). (Lucy) Carotenoid, Tocopherol, Phenolic Acid, and Antioxidant Properties of Maryland-Grown Soft Wheat. J. Agric. Food Chem..

[15] Tian W., Hu R., Chen G., Zhang Y., Wang W., Li Y. (2021). Potential Bioaccessibility of Phenolic Acids in Whole Wheat Products during in Vitro Gastrointestinal Digestion and Probiotic Fermentation. Food Chem..

[16] Tian W., Zheng Y., Wang W., Wang D., Tilley M., Zhang G., He Z., Li Y. (2022). A Comprehensive Review of Wheat Phytochemicals: From Farm to Fork and Beyond. Compr. Rev. Food Sci. Food Saf..

[17] Li D., Rui Y., Guo S., Luan F., Liu R., Zeng N. (2021). Ferulic Acid: A Review of Its Pharmacology, Pharmacokinetics and Derivatives. Life Sci..

[18] Liu J., Yu L.L., Wu Y. (2020). Bioactive Components and Health Beneficial Properties of Whole Wheat Foods. J. Agric. Food Chem..

[19] Ling Z., Xiao H., Chen W. (2022). Gut Microbiome: The Cornerstone of Life and Health. Adv. Gut Microbiome Res..

[20] Shewry P.R., Charmet G., Branlard G., Lafiandra D., Gergely S., Salgó A., Saulnier L., Bedő Z., Mills E.N.C., Ward J.L. (2012). Developing New Types of Wheat with Enhanced Health Benefits. Trends Food Sci. Technol..

[21] Tian W., Chen G., Gui Y., Zhang G., Li Y. (2021). Rapid Quantification of Total Phenolics and Ferulic Acid in Whole Wheat Using UV–Vis Spectrophotometry. Food Control.

[22] Tian W., Chen G., Zhang G., Wang D., Tilley M., Li Y. (2021). Rapid Determination of Total Phenolic Content of Whole Wheat Flour Using Near-Infrared Spectroscopy and Chemometrics. Food Chem..

[23] Tian W., Li Y. (2018). Phenolic Acid Composition and Antioxidant Activity of Hard Red Winter Wheat Varieties. J. Food Biochem..

[24] Li L., Shewry P.R., Ward J.L. (2008). Phenolic Acids in Wheat Varieties in the HEALTHGRAIN Diversity Screen. J. Agric. Food Chem..

[25] Zhang Y., Wang L., Yao Y., Yan J., He Z. (2012). Phenolic Acid Profiles of Chinese Wheat Cultivars. J. Cereal Sci..

[26] Hernandez-Espinosa N., Laddomada B., Payne T., Huerta-Espino J., Govindan V., Ammar K., Ibba M.I., Pasqualone A., Guzman C. (2020). Nutritional Quality Characterization of a Set of Durum Wheat Landraces from Iran and Mexico. LWT.

[27] Laddomada B., Durante M., Mangini G., D’Amico L., Lenucci M.S., Simeone R., Piarulli L., Mita G., Blanco A. (2017). Genetic Variation for Phenolic Acids Concentration and Composition in a Tetraploid Wheat (*Triticum turgidum* L.) Collection. Genet Resour Crop Evol.

[28] Tian W., Wilson T.L., Chen G., Guttieri M.J., Nelson N.O., Fritz A., Smith G., Li Y. (2021). Effects of Environment, Nitrogen, and Sulfur on Total Phenolic Content and Phenolic Acid Composition of Winter Wheat Grain. Cereal Chem..

[29] Barański M., Lacko-Bartošová M., Rembiałkowska E., Lacko-Bartošová L. (2020). The Effect of Species and Cultivation Year on Phenolic Acids Content in Ancient Wheat. Agronomy.

[30] Laddomada B., Blanco A., Mita G., D’Amico L., Singh R.P., Ammar K., Crossa J., Guzmán C. (2021). Drought and Heat Stress Impacts on Phenolic Acids Accumulation in Durum Wheat Cultivars. Foods.

[31] Ma D., Sun D., Li Y., Wang C., Xie Y., Guo T. (2015). Effect of Nitrogen Fertilisation and Irrigation on Phenolic Content, Phenolic Acid Composition, and Antioxidant Activity of Winter Wheat Grain. J. Sci. Food Agric..

[32] Tian W., Jaenisch B., Gui Y., Hu R., Chen G., Lollato R.P., Li Y. (2022). Effect of Environment and Field Management Strategies on Phenolic Acid Profiles of Hard Red Winter Wheat Genotypes. J. Sci. Food Agric..

[33] Tian W., Tong J., Zhu X., Martin P.F., Li Y., He Z., Zhang Y. (2021). Effects of Different Pilot-Scale Milling Methods on Bioactive Components and End-Use Properties of Whole Wheat Flour. Foods.

[34] Stumpf B., Yan F., Honermeier B. (2019). Influence of Nitrogen Fertilization on Yield and Phenolic Compounds in Wheat Grains (*Triticum aestivum* L. Ssp. Aestivum). J. Plant Nutr. Soil Sci..

[35] Kowalska I., Mołdoch J., Pawelec S., Podolska G., von Cossel M., Derycke V., Haesaert G., Lana M.A., da Silva Lopes M., Riche A.B. (2022). Environmental and Cultivar Variability in Composition, Content and Biological Activity of Phenolic Acids and Alkylresorcinols of Winter Wheat Grains from a Multi-Site Field Trial across Europe. J. Cereal Sci..

[36] Stumpf B., Yan F., Wen G., Eder K., Honermeier B. (2019). Dynamics of Antioxidant Properties, Phenolic Compounds, and Transcriptional Expression of Key Enzymes for the Phenylpropanoid Pathway in Leaves of Field-Grown Winter Wheat with Different Nitrogen Fertilization Schemes. J. Plant Nutr. Soil Sci..

